# How senior leaders support innovations in large learning health systems: Insights from United States Veterans Health Administration national program office leaders

**DOI:** 10.1002/lrh2.70012

**Published:** 2025-04-21

**Authors:** Jaifred Christian F. Lopez, Sallie Allgood, Kate Sheahan, Brandolyn White, M. Amy Kirshner, Suzanne Shirley, Madison Coffey, Amanda Milo, Sarah L. Cutrona, Laura Damschroder, Gemmae M. Fix, Andrea L. Nevedal, Caitlin M. Reardon, Marilla A. Opra Widerquist, Maria Arasim, Allen L. Gifford, Kathryn DeLaughter, George L. Jackson

**Affiliations:** ^1^ School of Medicine Duke University Durham North Carolina USA; ^2^ Center of Innovation to Accelerate Discovery and Practice Transformation Durham Veterans Affairs (VA) Health Care System Durham North Carolina USA; ^3^ Veterans Health Administration Innovation Ecosystem Richmond Virginia USA; ^4^ Rios Partners, LLC. Arlington Virginia USA; ^5^ Center for Health Optimization and Implementation Research VA Bedford Healthcare System Bedford Massachusetts USA; ^6^ Chan Medical School University of Massachusetts Worcester Massachusetts USA; ^7^ VA Ann Arbor Center for Clinical Management Research Ann Arbor Michigan USA; ^8^ Chobanian & Avedisian School of Medicine Boston University Boston Massachusetts USA; ^9^ Center for Health Optimization and Implementation Research, VA Boston Healthcare System Boston Massachusetts USA; ^10^ School of Public Health Boston University Boston Massachusetts USA; ^11^ Peter O'Donnell Jr. School of Public Health University of Texas Southwestern Medical Center Dallas Texas USA

**Keywords:** CFIR, implementation science, innovation, learning health system, qualitative, quality improvement, Veterans Health Administration

## Abstract

**Background:**

The U.S. Veterans Health Administration (VHA) formed an Innovation Ecosystem that develops and disseminates innovative practices to enhance Veterans' health. Support of senior leadership and their perception of the innovation process is key to the Ecosystem's success. We aimed to elicit insights on (1) how national VHA program office leaders define innovation, and (2) important considerations in facilitating the adoption of innovations.

**Methods:**

As part of a quality improvement initiative, we conducted 19 semi‐structured interviews via teleconference. Interviews involved 4 administration offices, 7 clinical and population health program offices, and 8 policy and quality improvement offices; 12 of these offices reported experience working with the Innovation Ecosystem. Responses were audio recorded, transcribed, and analyzed using constructs from the Consolidated Framework for Implementation Research.

**Results:**

Participants generally agreed that innovation within VHA is defined by evidence‐based development and implementation of interventions that improve response to Veterans' needs. Considerations in facilitating innovations include: (1) implementation climate that promotes network‐building, open communication, and well‐executed planning processes; (2) implementation infrastructures that enable engagement with key players and augment existing resources; and (3) innovation evidence strength and responsiveness to patient needs. Individuals working in policy‐related offices were more likely to identify complexity, leadership engagement, culture and available resources as factors in choosing innovations to adopt. Individuals who reported experience of working with the Ecosystem emphasized the importance of intra‐organizational networks and a favorable implementation climate, while those without experience noted the importance of working with external change agents. CFIR ‘inner setting’ constructs were seen in responses across all categories; meanwhile, emerging constructs highlight how innovation should be balanced by the reality of operations.

**Conclusions:**

Among national VHA program office leaders, innovation is pursued to improve Veterans' health. Resources, networks, culture, and processes are considered important factors among program office leaders to support and encourage innovation.

## 1 INTRODUCTION

The Veterans Health Administration (VHA) is the largest fully integrated health care system in the United States (U.S.), serving about 9.1 million enrolled Veterans. The VHA has four levels of organization: (1) VHA Central Office/Headquarters, led by the VA Under Secretary of Health, which includes a range of national program offices that provide overall leadership; (2) 18 Veterans Integrated Service Networks (VISNs) comprised of (3) 141 individual VA health care systems, and (4) 1254 locations including hospitals, clinics, and other care locations.^1^, ^2^

Administering care across such a large system has led to challenges that affect the quality of care.^3^ In 2014, quality concerns at VA facilities^4^, ^5^ served as the impetus for organizational and policy changes.^6^, ^7^ To support changes and enhance the diffusion of best practices across all levels and locations of the VHA, the VHA Innovation Ecosystem was developed in alignment with the U.S. National Academy of Medicine Learning Health System framework and its 2018 Future of Health Services report.^3^, ^8^, ^9^

### 1.1 The VHA Innovation Ecosystem

The VHA Innovation Ecosystem was created to support the development, replication, and spread of operationally focused innovations, many of which VHA employees developed. The Innovation Ecosystem is based out of the VHA Central Office, which provides leadership across all levels of the VHA. The Innovation Ecosystem works with other national program offices focused on research, innovation, and partnerships to support VHA's continued evolution as a learning health system towards the provision of the best possible care for Veterans.^10^, ^11^, ^12^, ^13^

VHA Innovation Ecosystem programs include the VHA Innovators Network (iNET) and VHA Diffusion of Excellence (DoE). iNET is a network of VHA facilities that host opportunities for frontline VHA employees seeking to develop innovative clinical and administrative practices.^14^, ^15^ The DoE program aims to foster the discovery and spread of mission‐driven health care innovations across VA^16^, ^17^, ^18^ and uses a “Shark Tank” mechanism to identify and support promising practices. DoE then facilitates the spread of successful innovations by providing dedicated resources and training.^16^, ^19^, ^20^ The Innovation Ecosystem also includes other efforts such as: a network of six National Centers for Innovation to Impact, Innovation Fellowships, the Community Engagement program, and the VHA Diffusion Marketplace website. With this portfolio, the VHA Innovation Ecosystem has created a pathway to support the innovation lifecycle from discovery to delivery.^11^, ^14^, ^16^, ^18^, ^19^, ^20^, ^21^, ^22^, ^23^, ^24^, ^25^, ^26^

### 1.2 VHA national program offices

The VHA Central Office/Headquarters includes national program offices (NPO) that provide operational and strategic direction to other levels within the VHA organization (e.g., VISN, healthcare systems, and individual facilities) and serve as partners for the nationwide spread of innovations. NPO leadership oversees some of the Nation's widest‐reaching health programs, focusing on a particular aspect of the healthcare delivery process (e.g., virtual care modalities), a specific health concern (e.g., mental health), or other cross‐cutting concerns (e.g., quality and performance).^27^ NPO leaders are well‐positioned to implement change and exercise a broader reach than that afforded to an individual medical facility or VHA region.^28^

However, NPO leaders across the VHA have varied responsibilities, areas of focus, and differing views on innovation. These differences can influence the way NPO leaders engage with researchers and innovation, and balance this with competing operational demands.^28^, ^29^ The success of the Innovation Ecosystem is dependent on alignment with NPO goals and a shared understanding of what it means to facilitate and support innovation. The goal of this quality improvement project was to understand how NPO leaders define the process of innovation and identify factors that influence leadership support of specific innovations.

## 2 METHODS

This quality improvement project was supported by the Spreading Healthcare Access, Activities, Research and Knowledge (SHAARK) Partnered Evaluation Initiative at the VHA.^18^, ^23^, ^25^, ^26^ Per regulations outlined in VHA Program Guide 1200.21, this evaluation has been designated a non‐research quality improvement activity. In order to frame understanding of the organizational, individual, and process‐related barriers and facilitators of implementation, the Consolidated Framework for Implementation Research (CFIR)^30^ was used to guide our analysis.

### 2.1 Evaluation design and implementation

This evaluation project was done with collaboration between VHA Innovation Ecosystem's Division of Community Engagement and Fellowships (IE‐CEF) and evaluators from SHAARK. Collaborators co‐developed the interview guide (guided by findings of previous SHAARK evaluations^20^, ^23^) and carried out purposive criterion sampling^31^ to capture the breadth of both NPOs and operational areas of collaboration with the Innovation Ecosystem. Inclusion criteria include: (1) appointment by the VA Central Office for at least 1 year, and (2) experience or interest in involvement with DoE activities, or in the planning, implementation, or supervision of an innovation coursed through the VHA Innovation Ecosystem. 30 individual leaders, representing 25 national program offices, were eligible for participation in interviews.^31^ These offices fell under one of three categories: (1) clinical or population health program offices, (2) policy and quality improvement offices, and (3) administrative, which oversees offices included in 1 and 2. Invitation emails were sent by VHA Innovation Ecosystem office representatives. IE‐CEF staff conducted interviews based on collaboratively developed plans, while SHAARK evaluators conducted the analysis.

Semi‐structured interviews were conducted through teleconference from February to April 2021. The approximately hour‐long interview included open‐ended questions addressing: (1) defining innovation; (2) facilitating innovation; (3) choosing which innovation to adopt within the organization; (4) supporting innovation within the organization; (5) infrastructure and other resources necessary to support innovation; and (6) experiences with DoE or the VHA Innovation Ecosystem. Interviews were conducted by the VHA Innovation Ecosystem's community engagement director (S.S.), with two management consultants serving as notetakers (M.C., A.M.). After securing informed consent, the interviews were audio recorded; participants who opted out of audio recording had their responses documented through structured notes.

Interviews were transcribed and coded by team members (K.S., B.W., M.K., G.J., S.A., and J.L.). Transcripts or structured notes were first coded to identify passages directly addressing each of the five questions. Next, using a directed qualitative content analysis approach (separately by at least two team members),^32^ transcripts or structured notes were coded using constructs of the CFIR.^30^ Codes were finalized by consensus. Emerging themes were also noted from passages that did not fit within a CFIR construct. Microsoft® Excel was used for code comparisons and pattern recognition.^33^

## 3 RESULTS

Twenty individual leaders participated: two individuals affiliated with the same office opted to participate in a joint interview session. Five of the 19 interview sessions involved the head of office accompanied by an assistant director or staff member; all other interviews were conducted solely with the head of office. A total of four administrative offices, seven clinical offices, and eight policy and quality improvement offices were included in this evaluation; respondent numbers from each office category are shown in Table 1. Ten offices were not able to participate due to schedule conflicts; nonetheless, after a preliminary analysis of the 19 interviews suggested data saturation, no further recruitment was done.^34^ A total of 12 interviews (63%) had participants reporting previous experience working with the Innovation Ecosystem or other related offices.

**TABLE 1:** Characteristics of offices interviewed (*n* = 19).

Type of national program office	Number of interviews^a^	Interviews with reported involvement with the VHA Innovation Ecosystem or related offices (%)	Interview numbers
Administrative	4	3 (75%)	1–4
Clinical/population health	7	3 (43%)	5–11
Policy and quality improvement	8	6 (75%)	12–19

^a^ There were interviews with more than one respondent (i.e., office lead or deputy with support staff): two out of four interviews with administrative offices, one out of seven clinical/population health, two out of eight policy and QI.

Salient CFIR constructs and exemplar quotes are presented in Tables 2 and 3, respectively, along with emerging themes (i.e., not covered sufficiently by any CFIR constructs).

**TABLE 2:** Overview of domains from the Consolidated Framework for Implementation Research (CFIR).^30^

Domain	Constructs used, definition
Intervention characteristics	*Evidence strength and quality*: Stakeholders' perceptions of the quality and validity of evidence supporting the belief that the intervention will have desired outcomes *Relative advantage*: Stakeholders' perception of the advantage of implementing the intervention versus an alternative solution *Complexity*: Perceived difficulty of implementation, reflected by duration, scope, radicalness, disruptiveness, centrality, and intricacy and number of steps required to implement
Outer setting	*Patient needs and resources*: The extent to which patient needs, as well as barriers and facilitators to meet those needs, are accurately known and prioritized by the organization
Inner setting	*Implementation climate*: The absorptive capacity for change, shared receptivity of involved individuals to an intervention, and the extent to which use of that intervention will be rewarded, supported, and expected within their organization. (Sub‐construct examples: tension for change, learning climate, and relative priority) *Readiness for implementation*: Tangible and immediate indicators of organizational commitment to its decision to implement an intervention (Sub‐construct examples: leadership engagement, available resources)
Process	*Planning*: The degree to which a scheme or method of behavior and tasks for implementing an intervention are developed in advance, and the quality of those schemes or methods *Engaging*: Attracting and involving appropriate individuals in the implementation and use of the intervention through a combined strategy of social marketing, education, role modeling, training, and other similar activities
Characteristics of individuals	(Constructs under this domain include knowledge and beliefs about the intervention, self‐efficacy, individual stage of change, individual identification with organization, and other personal attributes. No construct from this domain was used for this work.)

**TABLE 3:** Response categories and constructs observed from interview respondents, with exemplar quotes.

Response category	Constructs observed	Type of construct	Exemplar quotes
Defining innovation	Patient needs and resources	CFIR (outer setting)	“Diversity and inclusion [lead] all of us how to address the complicated issues and needs of Veterans… and be sure that we all understand what their needs are.”—#2
	Learning climate	CFIR (inner setting, implementation climate)	“[It is] finding new years of doing things that are outside the way we normally structure things.”—#3
	Relative advantage	CFIR (intervention characteristics)	“You want everyone to think this way: ‘Is there a better or different way to do things when you hit a wall? Is there another way to do what you're trying to do?’”—#5
	Evidence strength and quality	CFIR (intervention characteristics)	“Innovation… is taking evidence‐based services (i.e., things that have evidence behind them to support a benefit in terms of health promotion and disease prevention) and improving the reach of these to our veterans.”—#10
	Tension for change	CFIR (inner setting, implementation climate)	“[Part of it is] challenging the status quo to identify new ideas/processes—and continuing with this process; just because something was innovative does not mean it is still innovative.”—#4
	Multidimensionality of innovation	Emergent	“Innovation is multidimensional in terms of how it occurs in an organization committed to learning. Administrative and leadership innovations are just as critical as clinical innovations.”—#7
	Balancing research and operations	Emergent	“[I am] challenged with determining when an innovation is research and when it's not. If it's not research, then when should the operational side of innovation development work with research?”—#15
Facilitators of innovation	Networks and communications	CFIR (inner setting)	“Strong national and local networks that own the innovations delivered.”—#12
	Culture	CFIR (inner setting)	“We encounter everyday: ‘this is how we've always done it, why would we change it?’ We want to change that attitude and have folks look at big picture.”—#18
	Patient needs and resources	CFIR (outer setting)	“Through being part of the VHA modernization effort, which for the Office, is focusing on ways to help the whole person and their quality of life, moving beyond just their… symptoms.”—#8
	Leadership engagement	CFIR (inner setting, readiness for implementation)	“Communication from leadership encouraging, fostering, and supporting staff in being free to innovate, within a health system committed to learning…”—#7
	Local vs. broad‐scale innovation	Emergent	“Innovation at the local level should be encouraged and done so incrementally, i.e., not all at once, but bringing those innovations to scale should be prioritized with strategic thinking.”—#11
Choosing an innovation to adopt	Evidence strength and quality	CFIR (intervention characteristics)	“Having good metrics [is] important to show the effect and for us to have a way to sustain funding.”—#2
	Patient needs and resources	CFIR (outer setting)	“We can choose what is important based on themes that keep arising, e.g., how do we best care for an aging population of [Veterans]?”—#13
	Complexity	CFIR (intervention characteristics)	“Factors that are important is the project plan: scope (what we are trying to do, how big of an issue is it), goals and metrics ([we] want metrics to understand where gaps are but no so many that you can't cover things), evidence (some other place that has tested it, and VISNs that have tried it), risks (there may be some things that have to be dropped in order to develop the innovation).”—#5
	Compatibility	CFIR (inner setting, implementation climate)	“Innovations should build upon the existing systems/programs.”—#10
	Tension for change	CFIR (inner setting, implementation climate)	“Does it fulfill a need/gap?”—#11
	Relative priority	CFIR (inner setting, implementation climate)	“If it will ‘turn the dial’ and put VA in a different light and capacity to lead, not just in VA but in healthcare generally, then it is a go.”—#13
	Use of relevant data	Emergent	“If a researcher comes up with… an idea that [looks] at data that combines VA and community, I write off a letter of support instantaneously because that's a really, really good idea.”—#15
	Potential for impact	Emergent	“Look first at the problem we are trying to solve. How big is the problem? How many people does it impact? I like [big] impact.”—#13
	Working with researchers	Emergent	“Ongoing collaborations or involvement with research teams are important to adopting innovations.”—#10
Supporting innovation	Leadership engagement	CFIR (inner setting, readiness for iImplementation)	“Diffusion requires communication tools, change management, and buy‐in.”—#6
	Networks and communications	CFIR (inner setting)	“It would be helpful to have a mechanism that helps people with an innovative idea connect the dots across this huge organization to determine if that innovation already exists.”—#17
	Learning climate	CFIR (inner setting, implementation climate)	“It's important to keep people energized and coming up with ideas. How do we create ‘psychological safety’ so that people are not afraid of failure when innovating.”—#17
	Planning	CFIR (process)	“We have to devote time to making sure that everyone is on the same sheet of paper and talking about what ifs.”—#18
	Engaging	CFIR (process)	“Putting it up on a website or SharePoint site isn't going to be sufficient… How are we going to scale it, [and] convince people… how do we get the buy‐in to push things?”—#6
	Impact of feasibility assessments	Emergent	“When it comes to innovation, VA culture often leads to ‘on the fly’ feasibility assessment. This stifles innovation because it leads people to ask, ‘is this too hard or too bureaucratic to get this innovation in place?’ People may then assume that the innovation is unsupported or infeasible… We may stunt ourselves in this way because we think there are so many hurdles to the ideas, and we focus on that rather than on building on the ideas and moving them forward. We throw cold water on our ideas.”—#17
	Timelines	Emergent	“Timelines drive priorities of the organization and the budget.”—#5
	Bridging the research‐to‐practice gap	Emergent	“The research‐to‐practice timeline is often long and certainly the folks from QUERI, HSR&D and all the implementation science folks and even folks in informatics are helping flatten that. But then, getting it baked into practice because our providers are so busy, and they have to have a strong routine that enables them to get through their day effectively, and when we ask them to make a change in their routine, whether it's a progress note template… or something else to consider, I don't think we appreciate how disruptive that is, and how much we need to help them get that established.”—#8
	Ability to scale‐up	Emergent	“Centralized IT helps take promising innovations to scale.”—#4
	Navigating organizational requirements	Emergent	“There is a process for most things in the VA: what is the process for supporting innovation? Is the process a barrier? This process, whatever it is, should be standard across the organization. One way to categorize innovation is: “Is it a new market? A new technology? Or both?”—#11
Infrastructure for innovation	Networks and communications	CFIR (inner setting)	“[I am] seeing a real decline in collaboration and how we're able to do that idea sharing in real‐time; [the Innovation Ecosystem] may be able to help with that. [We] need training on how to do collaboration in a virtual environment.”—#3
	Available resources	CFIR (inner setting, readiness for implementation)	“Underlying infrastructure can limit what is possible in terms of innovation.”—#4
	Culture	CFIR (inner setting)	“[We] need a culture where innovators realize that regulations/policies/procedures can be altered as needed to meet innovation needs.”—#4
	External change agents	CFIR (process, engaging)	“Opportunities for multiple program offices to coalesce around ideas; [this] shows the value‐add of bringing offices together and makes for easier implementation.”—#7
	Relative priority	CFIR (inner setting, implementation climate)	“[There needs to be] shared alignment of a shared vision. We need to get better at identifying and clearly defining the innovations that show the most promise. Set parameters upfront to define what makes an innovation successful, and work throwing resources behind. Give a timeline for innovation testing. Make sure these innovations are things that match the vision, and focus on the most promising innovations, and stop focusing on innovation that does not show promise.”—#4
	Leadership engagement	CFIR (inner setting, readiness for implementation)	“Central/high level positions need to have a better understanding at the operational level.”—#16

### 3.1 Defining innovation

The process of innovation was defined by leaders and staff as the evidence‐based development and implementation of interventions aimed at improving the organization's response to Veteran health needs. The emphasis on responding to and learning from diverse patient needs and resources at the organizational level was shown by this response:

“Innovation [is part of being in a] learning organization that is committed to… implementing new things that the field can learn. [This can be seen in how we implement] diversity and inclusion: teaching all of us how to address the complicated issues and needs of Veterans who want to see people who look like them providing care… and be sure that we all understand what their needs are.”—Interviewee # 2 (#2), Administrative role

Innovation was seen as multidimensional, in that “administrative and leadership innovations are just as critical as clinical innovations.” (#7) Meanwhile, respondents also noted that innovation involves novelty through “thinking differently and beyond,” (#13) but also requires use of available technology and processes.

Notably, those who have had experience working with the Innovation Ecosystem saw the importance of an implementation climate and resources that foster continuous learning, as confirmed by one participant, who also raised questions about when it might be appropriate to engage with researchers.

“[Innovation involves the] grassroots process of finding, cultivating, and including industry partnerships. [However, we are] challenged with determining when an innovation is research and when it is not. If it's not research, then when should the operational side of innovation development work with research?”—#14, Policy/quality improvement role

### 3.2 Innovation facilitators

Program leaders/staff emphasized that what facilitates innovation is a leadership culture that is favorable to network‐building, open communication, and responsiveness to patient needs. One respondent provides an example of a VHA office that typifies this culture:

“They weave conversations about innovation into various collaboratives. They try to build an awareness that innovation and improvement are complementary. They leverage information and analytics innovatively to support VHA… and many of these are small innovations intended to make advancements within a system that wasn't designed to act in a particular way.”—#16, Policy/quality improvement role

Describing how leadership should communicate, a respondent stated that leaders should be “encouraging, fostering, and supporting staff in being free to innovate, [and staff should] feel safe and trusted to innovate.” (#7) The same respondent noted that leaders should also provide access to equitable funding and other resources to facilitate innovation. Other respondents emphasized that innovation is facilitated by “bringing people together, not 40 at once, but knowing who and when to bring in and out of the discussion,” (#5) and the capacity to bridge barriers related to regulations and policies, which is strengthened by having open communication that “[crosses] lines.” (#2) This aligns with the sentiments of those who have had experience working with the Innovation Ecosystem, who underline the importance of networks and a culture that encourages open communication within the organization; this is less emphasized by respondents that did not report such experience.

In some cases, program leaders/staff emphasized the need to navigate existing organizational requirements, how these can be leveraged to promote innovation, and how resulting practices are eventually included within routine operations.

“Can we optimize how we're delivering services once they get operational? …how can we have a sustainable practice to push out across our system?”—#6, Clinical role

### 3.3 Choice of innovation for adoption

Participants reported that an innovation was more likely to be adopted when it was an evidence‐based intervention responsive to patient needs, compatible with the organization, created tension for change, and was high priority to key players (cf. Table 2). One respondent noted: “innovation needs to have a clear pathway to integration and understanding if the VA has the tools to do it.” (#12) Another noted the importance of asking these questions:

“How big is the problem? How many people does it impact? Will this require system change or is it low hanging fruit? …what is the possibility for real change and improvement?”—#13, Policy/quality improvement role

Notably, some participants also spoke to the following emerging themes that influence the adoption of an innovation: use of locally relevant data, potential for impact, and the ability to work with researchers. Participants also noted that innovations are considered an investment and must be pitched with a strong business case.

“[The innovation needs to show] the cost benefit (ROI), fit with current environment and work demands, and clear measurable impact.”—#8, Clinical role.

### 3.4 Supporting innovation within organizations

Innovation can be well‐supported by an organizational climate that ensures the availability of resources, engagement of leaders and key players, functional networks and communication channels, and a well‐executed planning process.

“You have to spend a lot of time trying to convince people that the direction you're going is the right direction and they should put resources behind you and help you get there.”—#16, Policy/quality improvement role

Participants also described what supports innovation within organizations, including the timeline for innovation (and how this drives organizational priorities and budget), navigating organizational requirements, ease in upscaling, awareness of the need to bridge the research‐to‐practice gap, and the state of technology being used in the organization. Particularly:

“[There is] the need to flatten the research‐to‐practice timeline and translation thereof, [which is] often a long process. [There is also a] need to involve more end‐user participation and perspectives—innovations are often developed without a human‐centered design.”—#8, Clinical role

Respondents also highlighted the role of psychological safety:

“It's important to keep people energized and coming up with ideas, [and not throw] cold water on people's ideas because this will lead them to stop coming with ideas. How do we create psychological safety so that people are not afraid of failure when innovating, and indeed see failure as an opportunity?”—#17, Policy/quality improvement role

### 3.5 Infrastructure for innovation

Participants note that innovation is supported by infrastructure that improves facilities' networking and communication capacities, improves organizational culture (specifically in breaking down “siloes”), and promotes linkage with external change agents and engagement with leaders. Important considerations for innovation‐related infrastructure include the state of data infrastructure and organizational requirements that may need to be updated.

“Anything that can provide us with information that we can push and pull and [makes] it readily available and [eliminates] hours and hours of [typing] data into an email is invaluable to the organization and what we do.”—#18, Policy/quality improvement role

Respondents with experience of working with the Innovation Ecosystem emphasized that infrastructure is necessary to build and maintain networks and communication within the organization and foster a culture conducive to innovation. Meanwhile, those who did not report previous experience with the Innovation Ecosystem underlined the importance of linking with external change agents.

## 4 DISCUSSION

This work provides information on how innovation fits within a learning health system and contributes to our understanding of how program office goals can foster innovation within a health system. VHA national‐level leaders highlight that a novel idea alone is not sufficient for innovation success: additional requirements include addressing specific healthcare system challenges, as well as the extent to which it is feasible to implement and sustain a practice over the long run.

These insights build on previous work that analyzed factors that influence implementation and spread of specific ‘innovations’, which have been defined as “practices that improved the quality of health services offered within the VHA.”^35^ Meanwhile, in comparison to the Innovation Ecosystem's definition of innovation (i.e., ‘discovery operationalized to deliver impact’), the definition of innovation gathered from these interviews highlighted the importance of evidence‐based, needs‐based development of interventions, and was mostly aligned with the Ecosystem's definition. However, in defining innovation, participants also noted how innovation is made possible by an organizational climate that fosters quality improvement and is not averse to change, risk, or as one participant remarked, “stepping away from the status quo solution” (#6). These perspectives allude to the organizational aspect of innovation that is less emphasized by the Innovation Ecosystem's definition, which ostensibly focuses on the operationalization of evidence and its impact on patient care.

Insights generated from this work align with findings from similar evaluations. In one evaluation of facility leaders and staff undergoing training for implementation of the VHA Innovation Ecosystem, the following needs were elicited: ongoing alignment of operational priorities and capacity, recruitment and retention of implementation scientists and other specialists (especially those coming from underrepresented groups), and the need to sustain funding and data infrastructure to enable system‐wide research and quality improvement.^11^ Additionally, in a review on lessons learned from promoting research implementation in learning health systems, barriers identified in the research translation process include long timelines for research funding, limited relevance of research questions, lack of incentives to address real‐world settings, and lack of training in research methods.^28^, ^36^

Nonetheless, this work identified perspectives unique to national program office leaders. Firstly, due to their position within VHA and the hierarchical nature of government agencies in general, they were likely to encounter ‘siloes’ in working across VHA regions/VISNs and health facilities. Siloes, which pertain to sub‐organizational units with complex vertical hierarchies, have been found to hinder the ability to collaborate and share resources within organizations.^37^ Thus, these leaders are expected to benefit greatly from effective collaboration networks. Moreover, some participants acknowledged the need to address the research‐to‐practice gap, or the process of translating evidence into practices that become part of routine healthcare delivery, which is at the heart of VHA's commitment to become a learning health system but is often fraught with organizational challenges and lack of technical expertise.^10^, ^18^ In addition, as proponents of specific health programs, these national leaders need to demonstrate the value of their programs amid limited funding, equipment, and resources. Some responses underline the importance of pitching innovations as business cases, a skill that is honed by innovators through the VHA Shark Tanks; nonetheless, results of this evaluation suggest need of this skill within national program offices as well.

Furthermore, this work was conducted in 2021 during the coronavirus pandemic. A few leaders/staff mentioned that the pandemic forced health agencies to adopt innovative care solutions amid ensuing lockdowns and closures and led national program office leaders to revisit planning processes and prepare for unforeseen situations. Notably, in a related facility‐level study in 2020, the majority of surveyed facilities (70.7%) were still able to sustain the implementation of evidence‐informed practices, though a sizeable number of practices were put on hold due to public health guidelines in effect during that time.^26^ This suggests that individual facilities or health systems can continue implementing innovative practices amid health emergencies; nonetheless, an area for further evaluation is the role of national program office leaders in ensuring this sustainment.

In terms of CFIR constructs, results of this work align with current literature on how the inner setting influences the process of innovation. Responses confirm that aspects such as organizational climate, tension for change, leadership engagement, and collaborative networks positively influence innovation. Measures have been developed to assess the inner setting, which can be used in future evaluations.^38^ Meanwhile, responses also confirm the importance of intervention characteristics, especially relative advantage, alignment with current evidence, and alignment with organizational structure and ongoing operations. Responses also align with outer setting considerations, especially patient needs and resources, as well as process‐related aspects, such as planning and engaging with stakeholders.

Importantly, the emerging constructs point towards outcomes related to the implementation and innovation process. Respondents noted the need to reconfigure feasibility and impact assessments so that these do not appear to be ‘[throwing] cold water on [innovative ideas]’. A CFIR outcomes addendum was released in 2022 to address this gap.^39^ Among the emerging constructs, navigation of organizational requirements was a recurrent theme in responses related to supporting innovation. Using the lens of the 2022 outcomes addendum, these requirements seem to influence the anticipated implementation outcome of ‘adoptability,’ or ‘the likelihood that key decision makers will decide to put the innovation in place,’ or for ‘innovation deliverers [to] decide to deliver the innovation.’^39^ Recognizing these requirements may be key towards improved uptake of innovations: in an evaluation among innovation bidders participating in a VHA Shark Tank‐style competition, tools that aided bidders in determining facility needs, required resources, and compatibility facilitated the adoption of bids by individual facilities.^40^ Furthermore, emerging insights on scale‐up were also noted: a leader brought attention to how scale‐up at the local level should be encouraged, doing so ‘not all at once,’ but ‘[prioritizing] with strategic thinking.’ This aligns with findings from a 2020 evaluation in which the DoE's process for diffusion of innovative practices, with its strategic implementation support structure, has led to successful 6‐month implementation in more than 50% of participating facilities.^23^ Additionally, “centralized information technology” solutions were cited as facilitating the scale‐up of ‘promising innovations’; thus, solutions beyond the current VHA Diffusion Marketplace may need to be explored further.

These insights generally parallel evidence from other integrated health systems in the US and internationally and lend transferability^41^ to the results of this work. A challenge that is consistently noted is a cultural difference between those in charge of research and those leading clinical operations, thereby affecting how organizational priorities are set, which in turn influences general support and buy‐in.^29^, ^42^ Based on the results of this work, this challenge is seen to affect the capacity to address the health needs of Veterans: results suggest that addressing these needs will require reliable data infrastructure, health records, technical expertise on data analysis, and the capacity to act (and innovate accordingly) on data‐driven insights. As noted by participating leaders/staff, these tasks will require collaboration within the health system, especially between those in charge of research and those in clinical operations.

This work has important limitations. The representation of program office staff was not assured across all interviews; only five interviews included respondents other than the head or officer‐in‐charge of the national program office, thereby not providing the full range of ideas around innovation within VHA and specifically in participating in the VHA Innovation Ecosystem. The role of research in the innovation process, while cited by respondents, was not explored in depth and is beyond the scope of this work. Furthermore, due to the innovation‐oriented nature of this evaluation, individual leadership style, which is another potential factor that can influence the innovation process,^43^ was not considered in the design of this evaluation, nor was this brought up by participants' responses. Finally, this effort only focused on interviews with key representatives; it is anticipated that mixed methods work that includes other key players and analyzes other data (e.g., reports, administrative documents, and performance metrics), as well as other perspectives (e.g., individual leadership style) could help extend this work moving forward.

## 5 CONCLUSION

This evaluation shows that, among VHA national program office leaders, innovation is perceived as interventions aimed at improving response to the health needs of Veterans. To support and encourage innovation, these leaders consider the following as important: resources, networks, culture, and a process that includes prioritization, planning, and engagement with key players and change agents. Future evaluations may be enhanced by outcome‐based assessments, toward improvement of the process of innovation within health organizations.

## AUTHOR CONTRIBUTIONS

Jaifred Lopez led the drafting of the manuscript. Kate Sheahan, Brandolyn White, Sallie Allgood, Amy Kirshner, Suzanne Shirley, Madison Coffey, and Amanda Milo coordinated with national leaders/staff, conducted the interviews, and did the transcripts. George Jackson, Brandolyn White, Kate Sheahan, Sallie Allgood, and Jaifred Lopez developed the codebook and implemented the analysis approach, with technical and editorial input from Laura Damschroder, Sarah Cutrona, Gemmae Fix, Andrea Nevedal, Caitlin Reardon, Allen Gifford, Maria Arasim, Marilla Widerquist, and Kathryn DeLaughter, all of whom contributed to the completion of the manuscript. George Jackson, Laura Damschroder, Sarah Cutrona, Gemmae Fix, Andrea Nevedal, Caitlin Reardon, Kathryn DeLaughter, and Allen Gifford were major contributors in the development of the VHA Innovation Ecosystem partnered evaluation and its analysis frameworks. All authors read and approved the final manuscript.

## FUNDING INFORMATION

This evaluation is supported by the VA Quality Enhancement Research Initiative (QUERI) and Health Services Research & Development Service (grants: PEC 17‐002; QUE 20‐012; SDR 20‐389; CIN 13‐410) and VHA Office of Rural Health through the VHA Diffusion of Excellence/Innovation Ecosystem.

## CONFLICT OF INTEREST STATEMENT

All authors are or have been affiliated with the US Department of Veterans Affairs. No author received compensation for preparation of this manuscript apart from his or her employment.

## ETHICS STATEMENT

Per regulations outlined in VHA Program Guide 1200.21, this evaluation has been designated a non‐research quality improvement activity and has been exempt from ethics review.

## DISCLAIMER

The views expressed in this paper are those of the authors and do not reflect the position or policy of the Department of Veterans Affairs, the United States government, or other organizations with which the authors are affiliated.

## Data Availability

The anonymized datasets used and/or analyzed are potentially available from the corresponding author on reasonable request and agreement of the VHA Innovation Ecosystem/Office of Healthcare Innovation and Learning.
